# 575. Trial by Fire: Insights from the Frontlines of a Burn Disaster Response

**DOI:** 10.1093/jbcr/irag033.338

**Published:** 2026-04-06

**Authors:** Emily Hagg, Danielle H Fuchko, Vincent Gabriel

**Affiliations:** Alberta Health Services, Calgary, AB; Alberta Health Services, Calgary, AB; University of Calgary, Calgary, AB

## Abstract

**Introduction:**

When an urban residential explosion resulted in seven burn injured patients presenting simultaneously to a Level 1 trauma and burn centre, an all-hazards mass casualty incident response plan was activated. All patients were managed at a single burn centre, which was overcapacity at the time of the incident. To inform the creation of a burn-specific disaster response plan, the authors sought to understand what occurred during the health system response to a burn mass casualty incident, what strengths and issues were identified, and how these could be addressed in future incident response plans.

**Methods:**

Ethics Board and organizational approvals were obtained. An anonymous 15-question digital REDCap survey was sent to management or administrative support personnel in the Emergency Department, Intensive Care Unit, Inpatient Burn Unit, and Outpatient Burn Clinic. These intermediaries then distributed the survey via email to their staff with the request for responses from those involved in the specified incident. Two reminders were sent out to ensure responses were collected from each department. Questions were multiselect with an option for free text answer. Thematic analysis of free text answers was completed by all three authors independently and compared for interrater reliability. Thematic consensus was obtained.

**Results:**

Sixteen responses were collected across the multidisciplinary team (Fig. 1). Participants were evenly distributed across the spectrum of length of time involved in the incident response (Table 1). Respondents indicated strong initial response capacity, with minimal/nonexistent challenges identified in the earliest phases. However, supply availability, staffing, and physical space were challenging in the weeks to months phase. The lack of recognition of the ongoing challenges faced by staff weeks to months following the incident was highlighted as a concern. However, across all phases, teamwork was consistently rated highly.

**Conclusions:**

Effective early-phase response systems are critical and were well-supported. Resource allocation and infrastructure support diminished significantly in prolonged care settings. Therefore, long-term care phases require improved interdisciplinary planning, resources, and communication strategies. Collaboration is a resilient asset across the continuum of care.

**Applicability of Research to Practice:**

Findings of this study may inform a robust review of burn centres’ mass casualty incident response plans with focus on improving prolonged response strategies.

**Funding for the study:**

REDCap survey services funded by an unrestricted philanthropic grant.

